# Prognostic value of upper respiratory tract microbes in children presenting to primary care with respiratory infections: A prospective cohort study

**DOI:** 10.1371/journal.pone.0268131

**Published:** 2022-05-12

**Authors:** Luke J. McGeoch, Hannah V. Thornton, Peter S. Blair, Hannah Christensen, Nicholas L. Turner, Peter Muir, Barry Vipond, Niamh M. Redmond, Sophie Turnbull, Alastair D. Hay

**Affiliations:** 1 Population Health Sciences, Bristol Medical School, University of Bristol, Bristol, United Kingdom; 2 Centre for Academic Primary Care, Population Health Sciences, Bristol Medical School, University of Bristol, Bristol, United Kingdom; 3 Bristol Randomised Trials Collaboration, Bristol Trials Centre, University of Bristol, Bristol, United Kingdom; 4 NIHR Health Protection Research Unit in Behavioural Science and Evaluation, Bristol Medical School, University of Bristol, Bristol, United Kingdom; 5 South West Regional Laboratory, National Infection Service, Public Health England, Bristol, United Kingdom; 6 Centre d’épidémiologie et de recherche en santé des populations (CERPOP), Université Toulouse III—Paul Sabatier, Toulouse, France; Carol Davila University of Medicine and Pharmacy: Universitatea de Medicina si Farmacie Carol Davila, ROMANIA

## Abstract

**Background:**

The association between upper respiratory tract microbial positivity and illness prognosis in children is unclear. This impedes clinical decision-making and means the utility of upper respiratory tract microbial point-of-care tests remains unknown. We investigated for relationships between pharyngeal microbes and symptom severity in children with suspected respiratory tract infection (RTI).

**Methods:**

Baseline characteristics and pharyngeal swabs were collected from 2,296 children presenting to 58 general practices in Bristol, UK with acute cough and suspected RTI between 2011–2013. Post-consultation, parents recorded the severity of six RTI symptoms on a 0–6 scale daily for ≤28 days. We used multivariable hurdle regression, adjusting for clinical characteristics, antibiotics and other microbes, to investigate associations between respiratory microbes and mean symptom severity on days 2–4 post-presentation.

**Results:**

Overall, 1,317 (57%) children with complete baseline, microbiological and symptom data were included. Baseline characteristics were similar in included participants and those lacking microbiological data. At least one virus was detected in 869 (66%) children, and at least one bacterium in 783 (60%). Compared to children with no virus detected (mean symptom severity score 1.52), adjusted mean symptom severity was 0.26 points higher in those testing positive for at least one virus (95% CI 0.15 to 0.38, p<0.001); and was also higher in those with detected Influenza B (0.44, 0.15 to 0.72, p = 0.003); RSV (0.41, 0.20 to 0.60, p<0.001); and Influenza A (0.25, -0.01 to 0.51, p = 0.059). Children positive for Enterovirus had a lower adjusted mean symptom severity (-0.24, -0.43 to -0.05, p = 0.013). Children with detected *Bordetella pertussis* (0.40, 0.00 to 0.79, p = 0.049) and those with detected *Moraxella catarrhalis* (-0.76, -1.06 to -0.45, p<0.001) respectively had higher and lower mean symptom severity compared to children without these bacteria.

**Conclusions:**

There is a potential role for upper respiratory tract microbiological point-of-care tests in determining the prognosis of childhood RTIs.

## Introduction

Globally, upper and lower respiratory tract infections (RTIs) are frequent reasons for primary care attendance and are more common in young children and older adults [1–3]. Whilst mortality rates have declined in recent decades, RTIs continue to be a major cause of morbidity in children [1,2]. More developed settings have the highest age-standardised incidence rates and disability-adjusted life year (DALY) rates for upper RTIs [1,2]. In the UK, primary care presentations with RTIs are more common in urban and in more deprived areas [4]. The majority of childhood RTIs are caused by respiratory viruses and can be safely managed conservatively [5]. The vast majority of symptoms typically resolve within two weeks [6]. Despite this, around half of childhood RTIs are treated with antibiotics [7], of which half are unnecessary [8]. Further, RTIs account for the majority of inappropriate antibiotic prescribing in primary care [8]. This contributes to the development of antimicrobial resistance and the potential for infections to become untreatable in the future [9]. Inappropriate antibiotic prescribing also incurs economic costs and a risk of adverse effects [5].

Clinical signs and symptoms alone, of which acute cough is the most common, do not predict clinical prognosis or the presence of viruses or bacteria [10,11]. Therefore, they are a poor guide to the likely severity of illness or appropriateness of antibiotic prescribing, resulting in clinical uncertainty [12]. An antimicrobial resistance review commissioned by the UK Department of Health and Social Care advocated the use of rapid diagnostics to reduce unnecessary antibiotic prescribing [13]. Consequently, microbiological point-of-care tests (POCTs) of the nasopharynx have garnered increasing interest in relation to RTIs, on the basis that they may limit antibiotic prescribing to susceptible bacterial infections [12]. Despite use in hospital settings and advancements made during the COVID-19 pandemic, microbiological POCTs have traditionally not been used in primary care settings. This reflects long run times, an inability to test for a diverse range of microbes, high costs, and insufficient evidence [12]. However, we recently demonstrated the feasability and acceptability of a POCT based on real-time nested polymerase chain reaction (PCR). This POCT tested for 17 respiratory viruses and three atypical bacteria in patients aged ≥3 months presenting to the primary care setting with suspected RTI. It had a turnaround time of 65 minutes [12]. Nevertheless, it remains the case that little is known about the diagnostic and prognostic significance of microbes detected from the upper respiratory tract [11]. The respiratory tract harbours a range of microorganisms, which may not necessarily cause infection. One way to distinguish pathogenic from commensal microbes is to investigate whether the presence of microbes influences disease prognosis. Knowledge of this relationship could inform care and safety netting recommendations to parents. There is a paucity of evidence for this in children [3]. Therefore, the objective of the present study was to use an existing dataset to investigate the prognostic significance of upper respiratory tract microbes in children presenting to primary care with RTIs.

## Methods

### Study population

The population included was a subgroup of the ‘TARGET’ study, a cohort of 8,394 children aged between 3 months and up to 16 years presenting to one of 247 general practices between July 2011 and May 2013. Children were eligible for the ‘TARGET’ study if they presented with a RTI with cough of ≤28 days duration prior to consultation. Other RTI symptoms may or may not have been present at study baseline. Children were excluded if they presented with a non-infective exacerbation of asthma, with RTI without cough, or with cough of >28 days duration. Children were also excluded if they were considered to have a high risk of serious infection, or they required a throat swab as part of normal clinical care. Full eligibility criteria are outlined in the TARGET study protocol [14]. Recruitment was coordinated by centres in Bristol, London, Oxford and Southampton, and study methods and main results have been reported [14,15].

Here we report results from 2,296 children recruited to the Bristol centre who, in addition to the baseline demographic and clinical data collected by parents and general practitioners or nurse practitioners using a structured proforma (S1 Fig), were invited to provide a pharyngeal swab and for parents to complete a daily symptom diary. The final analytic sample in this study consisted of children with complete microbiological data and symptom diary data for two or more of days 2–4 post-consultation. This interval was selected as the period when symptoms are usually most severe [16,17], and close enough to the microbiological sampling time to be considered relevant.

### Microbiological samples and processing

As reported previously [11], general and nurse practitioners received training in taking samples [14]. This involved sweeping a dual polyurethane foam tipped swab (Medical Wire and Equipment, Corsham, UK) across the mucus membranes of the posterior oropharynx, with instructions to touch both tips on both sides of the throat. The swab tips were snapped off and sealed into separate plastic specimen vials containing transport medium (∑-Virocult® transport medium for virological testing and ∑-Transwab® liquid Amies medium for bacteriological testing). Vials were transported either by first-class Post Office Safebox™ or existing same-day hospital transport to the Bristol Centre for Antimicrobial Research and Evaluation (BCARE) at Southmead Hospital, Bristol, UK. The bacterial culture laboratory processed one vial and sent the second to the viral laboratory for identification of viruses and additional bacteria by semi-quantitative real-time PCR. Samples were amplified and analysed using a Custom TaqMan low-density array (TLDA) system on an Applied Biosystems^TM^ Life Technologies ViiA-7 real-time PCR system (Thermo Fisher Scientific Inc.). Agar plates were incubated overnight and colonies morphologically consistent with the following eight microbes were identified using standard laboratory tests: *Haemophilus influenzae*, *Moraxella catarrhalis*, *Staphylococcus aureus*, beta haemolytic Streptococci (Groups A, C, F, G), and *Streptococcus pneumoniae*. Viruses and bacteria detected using PCR included Adenovirus, Bocavirus, Coronavirus (types 229E, NL63, OC43, OC43/HKU1), Enterovirus, Influenza A and B, Metapneumoviruses, Parainfluenzavirus (types 1–4), Parechovirus, Respiratory Syncytial Virus (RSV) (types A and B), Rhinovirus, *Bordetella pertussis*, *Bordetella parapertussis*, *Chlamydia pneumoniae*, and *Mycoplasma pneumoniae*. A cycle threshold (Ct) value ≤35 was chosen as the cut-off for a positive result.

### Prognostic outcome

As previously reported [18], parents were invited to complete a validated [19] symptom diary. This has previously been shown to accurately predict the proportion of children recovering within two weeks [20]. Parents completed either a paper-based or online version of the diary depending on personal preference. The severity of six symptoms (cough, shortness of breath, impaired sleep, being unwell, reduced level of activity, and temperature) were recorded once daily using a 7-item Likert scale from zero (‘normal’) to six (‘as bad as it could be’). Parents were asked to continue recording for up to 28 days post-consultation, or until all symptoms were scored ‘normal’ for two consecutive days, where ‘day 1’ was the day of consultation and recruitment. Study team administrators telephoned parents to support diary completion up to 3 times during the first 7 days and then at least once per week until day 28 if no data had been obtained that week. Text messages and postcard reminders were also used to maximise diary completion and return. All diaries were checked for accuracy and completion Two attempts were made to contact parents to clarify any data queries. Scores were converted into a mean symptom score across the six symptoms for days 2, 3 and 4 (mSS_2-4_). To create the mSS_2-4_ outcome, we took the mean severity for each symptom across days 2–4 post-consultation, then took the mean of these scores across all symptoms, giving a final value on a 0 to 6 scale. Where children were missing symptom data for a single day of days 2–4 (n = 27), mSS_2-4_ was calculated using the data for the two days with complete data. As indicated in the original study protocol, microbiological sample results were not made available to recruiting clinicians, since the clinical significance of the results had not been established [14].

### Statistical analysis

Baseline characteristics were described using means and standard deviations (SD) for normally distributed continuous variables, medians and interquartile ranges (IQR) for non-normally distributed continuous variables, and frequencies and percentages for categorical variables.

The relationship between positivity for one or more respiratory viruses or bacteria, and mSS_2-4_, fitted as a continuous variable, was computed using a linear Cragg hurdle regression model [21,22]. This model combines a selection model, determining whether an individual has a mean symptom score of zero (asymptomatic), with an outcome model investigating the relationship between the presence or absence of viruses or bacteria and mSS_2-4_ among individuals with non-zero values. This model was selected to account for zero-inflation of symptom scores. Both univariable and multivariable models were used to compute differences in mSS_2-4_ and corresponding 95% confidence intervals (CIs) and p-values. In analyses exploring the relationship between positivity for one or more viruses or bacteria, and mSS_2-4_, the reference group consisted of participants testing negative for all corresponding microbes. In analyses examining potential associations with individual viruses or bacteria, the reference group consisted of participants testing negative for the corresponding virus or bacterium.

Potential covariates considered on grounds of clinical importance were: age, sex, ethnicity, index of multiple deprivation (IMD) score, clinician-reported baseline disease severity, presence of chronic disease, antibiotic prescribing, illness duration preceding study recruitment, time taken for swabs to be received in the laboratory, and for virus models and bacteria models, positivity for one or more bacteria or viruses respectively. Covariates were selected for inclusion in the multivariable models using a threshold p-value of 0.10 following addition to a model examining the association between positivity for one or more viruses or bacteria and mSS_2-4_. This followed the approach outlined in the TARGET study protocol [14]. Models with individual viruses or bacteria as the primary predictor were additionally adjusted for the presence of co-infection with one or more other viruses or bacteria respectively.

All statistical testing was two-sided. All analyses were conducted using Stata/MP version 16.1 [23].

### Ethical approval

The study was approved by the South West Central Bristol Research Ethics Committee, UK (reference number 10/H0102/54). Research governance approvals were obtained for all areas before the start of recruitment. Written informed consent was obtained from one parent (and assent from children aged ≥11 years). The study was conducted in accordance with the principles expressed in the Declaration of Helsinki.

## Results

### Baseline characteristics

**Fig 1:**
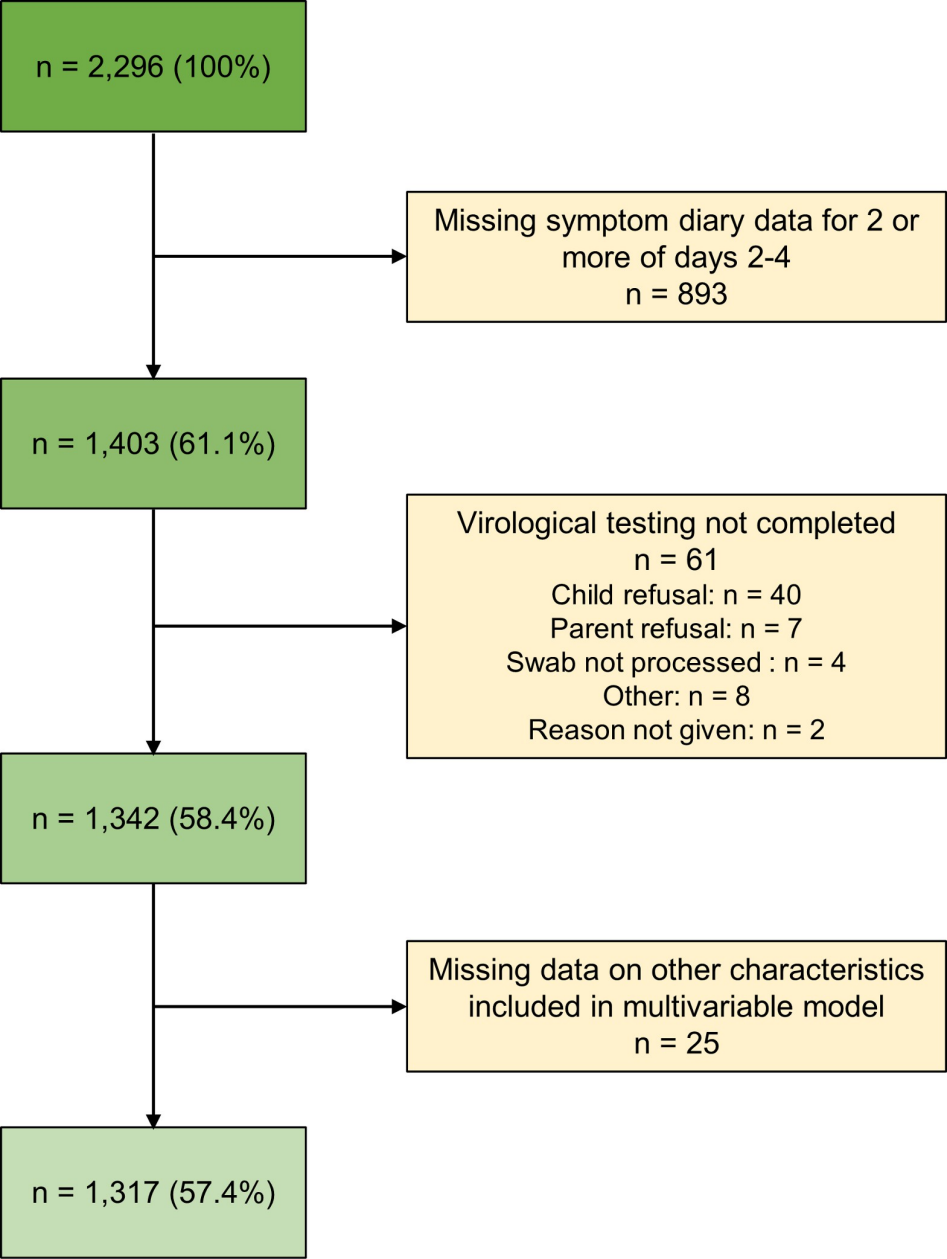
Flow chart of participants through the study.

Of the 2,296 children recruited by the Bristol centre, 893 (39%) were excluded due to incomplete symptom score data for two or more of days 2–4. A further 61 and 25 children respectively had incomplete virological and baseline characteristic data, leaving 1,317 participants contributing to the analysis (Fig 1).

Baseline characteristics of included and excluded children were similar, except that excluded children were from more deprived households (S1 Table).

Table 1 presents the characteristics of the participants testing positive (869; 66%) and negative (448; 34%) for one or more respiratory virus. Compared to those testing negative for all viruses, children testing positive were younger (mean 4.3 vs. 5.8 years); presented sooner after illness onset (median 5 (IQR 3–9) vs. 7 (4–14) days); and clinicians reported them to be slightly more unwell (median severity score 3/10 (2–4) vs. 2/10 (1–4)). They were similar with respect to sex, ethnicity, and antibiotic prescribing. Compared to those testing negative for bacteria, children testing positive were older (mean 5.1 vs. 4.4 years) and more likely to test positive for viruses (70% vs. 60%) (S2 Table).

**Table 1:** Baseline characteristics of children stratified by the presence of any virus detected^a^.

	Virus (n = 869)	No virus (n = 448)
**Mean age (years, SD)**	4.28 (3.82)	5.82 (4.33)
**Male, n (%)**	472 (54.3)	222 (49.6)
**White, n (%)**	771 (88.7)	411 (91.7)
**IMD decile** * **, n (%)**		
Least deprived 1	85 (9.8)	48 (10.7)
2	97 (11.2)	62 (13.8)
3	67 (7.7)	38 (8.5)
4	100 (11.5)	35 (7.8)
5	96 (11.1)	36 (8.0)
6	108 (12.4)	50 (11.2)
7	70 (8.1)	44 (9.8)
8	81 (9.3)	41 (9.2)
9	87 (10.0)	42 (9.4)
Most deprived 10	78 (9.0)	52 (11.6)
**History of chronic disease** ^†^ **, n (%)**	145 (16.7)	111 (24.8)
**Median number of days of illness before presentation (IQR)**	5 (3–9)	7 (4–14)
**Median clinician-reported Illness severity at consultation, score (IQR)**	3 (2–4)	2 (1–4)
**Antibiotic prescribed at consultation, n (%)**	331 (38.1)	178 (40.0)
**Re-consultation within 30 days, n (%)**	198 (22.8)	101 (22.5)
**Hospitalisation within 30 days, n (%)**	15 (1.7)	3 (0.7)
**Baseline symptoms, n (%)**		
Dry cough	449 (51.7)	248 (55.4)
Productive cough	519 (59.7)	266 (59.4)
Barking cough	272 (31.3)	126 (28.1)
Blocked/runny nose	718 (82.6)	295 (65.9)
Change in cry	187 (21.6)	53 (11.9)
Shortness of breath	385 (44.3)	161 (36.0)
Wheeze	403 (46.4)	187 (41.7)
Fever	556 (64.0)	238 (53.1)
Shivering	224 (25.8)	112 (25.0)
Diarrhoea	127 (14.6)	52 (11.6)
Vomiting	261 (30.0)	114 (25.5)
Reduced fluid intake	283 (32.6)	83 (18.5)
Reduced eating	564 (64.9)	233 (52.0)
Low energy	461 (53.1)	218 (48.7)
Disturbed sleep	698 (80.3)	343 (76.6)
Reduced urine output	136 (15.7)	38 (8.5)
**Baseline clinical signs, n (%)**		
Elevated pulse	51 (5.9)	31 (7.0)
Wheeze	160 (18.4)	87 (19.4)
Crackles	179 (20.6)	104 (23.2)
Bronchial Breathing	20 (2.3)	6 (1.3)
**Number of viruses present, n (%)**		
1	631 (72.6)	-
2	204 (23.5)	-
3	30 (3.5)	-
4	4 (0.5)	-
**Number positive for individual viruses, n (%)**		
Rhinovirus	363 (41.8)	-
RSV^‡^	135 (15.5)	-
Enterovirus	123 (14.2)	-
Parainfluenzae^§^	119 (13.7)	-
Influenza A	75 (8.6)	-
Adenovirus	70 (8.1)	-
Coronavirus^¶^	62 (7.1)	-
HMPV	62 (7.1)	-
Influenza B	61 (7.0)	-
Bocavirus	50 (5.8)	-
Parechovirus	22 (2.5)	-
**Bacteria**^**b**^ **detected, n (%)**	548 (63.1)	235 (52.5)

* The English Indices of Multiple Deprivation 2007 score, linked to the child’s postcode, was used as a surrogate for home deprivation.

^†^ Chronic diseases were defined as asthma, bronchiectasis, cystic fibrosis, diabetes, epilepsy, HIV, splenectomy, or ‘other’.

^‡^ Types A and B.

^§^ Types 1–4.

^¶^ Types 229E, NL63, OC43, OC43/HKU1.

^a^ Since the presence of bacteria was not associated with mean symptom severity on days 2–4.

^b^ Bacteria tested were *Bordetella pertussis*, *Bordetella parapertussis*, *Chlamydia pneumoniae*, *Haemophilus influenzae*, *Moraxella catarrhalis*, *Staphylocococcus aureus*, *Mycoplasma pneumoniae*, beta haemolytic Streptococci (Groups A, C, F, G), and *Streptococcus pneumoniae*.

Abbreviations: SD, standard deviation; IMD, index of multiple deprivation; IQR, interquartile range; RSV, Respiratory Syncytial Virus; HMPV, Human Metapneumovirus.

### Microbes detected

Of the children testing positive for respiratory viruses, the majority (72.6%) tested positive for a single virus, with 23.5% testing positive for two viruses, 3.5% for three viruses and 0.5% for four viruses (Table 1). The most detected viruses among all included participants were Rhinovirus (28%), RSV (10%), Influenza A or B (collectively detected in 10%), Enterovirus (9%) and Parainfluenzae Viruses (9%). Bacteria were more commonly detected among children who tested positive for viruses compared to those who tested negative (63% vs. 53%). The most detected bacteria among all included participants were *Staphylococcus aureus* (33%), *Haemophilus influenzae* (24%), *Streptococcus pneumoniae* (15%) and Group A beta-haemolytic streptococci (8%) (S2 Table). 548 (41.6%) children simultaneously tested positive for at least one virus and at least one bacterium.

Overall younger children were more likely to test positive for viruses, whereas older children were more likely to test positive for bacteria. However, the age distribution of positive tests varied for individual organisms. For instance, positivity for influenza viruses was more common among older children and positivity for *S*. *pneumoniae* was more common among younger children (S3 Table).

### Mean symptom score days 2–4

Among children with no virus detected, mean mSS_2-4_ was 1.52 (SD 0.97) (Table 2). In the unadjusted analysis, compared to those children in whom no virus was detected, a higher mean mSS_2-4_ was reported among children testing positive for one or more viruses (difference 0.23 points, 95% CI 0.11 to 0.34, p<0.001). Since all potential covariates, with the exception of positivity for one or more bacteria (in the virus model) and time taken for swabs to reach the laboratory, were associated with mSS_2-4_ (at p<0.10), they were included in the final multivariable models. Positivity for one or more bacteria was also included in the virus model in view of its putative clinical significance. Following adjustment for covariates, there remained evidence of a higher mean mSS_2-4_, with a difference of 0.26 points (0.15 to 0.38, p<0.001). We also identified evidence in fully adjusted analyses that the detection of two viruses were associated with an increased mean mSS_2-4_: Influenza B (0.44, 0.15 to 0.72, p = 0.003) and RSV (0.41, 0.20 to 0.61, p<0.001). There was weak evidence of an association for Influenza A: 0.25 (-0.01 to 0.51, p = 0.059) The detection of Enterovirus was associated with a lower mean mSS_2-4_ (-0.24, -0.43 to -0.05, p = 0.013).

**Table 2:** Mean symptom severity between days 2–4 after initial illness presentation among children testing positive for respiratory viruses.

	N	Mean symptom score (SD)	Unadjusted difference between groups (95% CI)	P-value	Adjusted for presence of other viruses (95% CI)	P-value	Fully adjusted* (95% CI)	P-value
**No virus present**	448	1.52 (0.97)	Ref	Ref	Ref	Ref	Ref	Ref
**≥1 virus**	869	1.75 (1.06)	0.23 (0.11 to 0.34)	<0.001	-	-	0.26 (0.15 to 0.38)	<0.001
**Influenza B**	61	2.20 (1.15)	0.56 (0.27 to 0.85)	<0.001	0.57 (0.28 to 0.87)	<0.001	0.44 (0.15 to 0.72)	0.003
**RSV** ^**†**^	135	2.02 (1.06)	0.39 (0.20 to 0.58)	<0.001	0.37 (0.18 to 0.56)	<0.001	0.41 (0.20 to 0.60)	<0.001
**Influenza A**	75	1.97 (1.14)	0.32 (0.06 to 0.59)	0.016	0.30 (0.05 to 0.57)	0.021	0.25 (-0.01 to 0.51)	0.059
**Adenovirus**	70	1.88 (1.08)	0.23 (-0.03 to 0.48)	0.084	0.18 (-0.09 to 0.45)	0.193	0.21 (-0.06 to 0.48)	0.130
**HMPV**	62	1.90 (1.19)	0.24 (-0.06 to 0.54)	0.115	0.21 (-0.09 to 0.51)	0.168	0.20 (-0.10 to 0.50)	0.186
**Parainfluenzae** ^**‡**^	119	1.76 (1.02)	0.10 (-0.09 to 0.29)	0.307	0.07 (-0.12 to 0.27)	0.466	0.10 (-0.10 to 0.29)	0.332
**Bocavirus**	50	1.70 (1.11)	0.04 (-0.27 to 0.35)	0.807	-0.03 (-0.34 to 0.28)	0.850	0.06 (-0.26 to 0.38)	0.707
**Parechovirus**	22	1.55 (0.94)	-0.12 (-0.50 to 0.27)	0.556	-0.19 (-0.56 to 0.19)	0.327	-0.08 (-0.45 to 0.30)	0.678
**Rhinovirus**	363	1.62 (1.03)	-0.06 (-0.19 to -0.07)	0.355	-0.11 (-0.24 to 0.02)	0.111	-0.12 (-0.25 to 0.01)	0.064
**Coronaviruses** ^**§**^	62	1.59 (0.97)	-0.08 (-0.33 to 0.17)	0.522	-0.13 (-0.37 to 0.12)	0.318	-0.12 (-0.36 to 0.12)	0.312
**Enterovirus**	123	1.51 (0.96)	-0.18 (-0.36 to 0.00)	0.052	-0.28 (-0.47 to -0.10)	0.003	-0.24 (-0.43 to -0.05)	0.013

The reference group for analyses investigating the relationship between presence or absence of a single virus and mean symptom score was absence of the corresponding virus.

* Model adjusted for age, sex, ethnicity, index of multiple deprivation, clinician-reported baseline illness severity, duration of illness preceding presentation, presence of chronic disease, whether antibiotic prescribed at time of presentation, and presence of bacteria. Models including individual viruses were additionally adjusted for presence of one or more other viruses.

^†^ Types A and B.

^‡^ Types 1–4.

^§^ Types 229E, NL63, OC43, OC43/HKU1.

Among children with no bacterium detected, mean mSS_2-4_ was 1.66 points (SD 1.04) (Table 3). Compared with these children, mean mSS_2-4_ was not different in children with any bacterium detected in univariable (difference 0.02 points, -0.09 to 0.14, p = 0.715) or multivariable (-0.02, -0.14 to 0.09, p = 0.688) analyses. The detection of two bacteria were related to differences in mean mSS_2-4_ in adjusted analyses: *Moraxella catarrhalis* (-0.76, -1.06 to -0.45, p<0.001) and *Bordetella pertussis* (0.40, 0.00 to 0.79, p = 0.049).

**Table 3:** Mean symptom severity between days 2–4 after initial illness presentation among children testing positive for respiratory bacteria.

	N	Mean symptom score (SD)	Unadjusted difference between groups (95% CI)	P-value	Adjusted for presence of other bacteria (95% CI)	P-value	Fully adjusted* (95% CI)	P-value
**No bacteria present**	534	1.66 (1.04)	Ref	Ref	Ref	Ref	Ref	Ref
**≥1 bacterium**	783	1.68 (1.04)	0.02 (-0.09 to 0.14)	0.715	-	-	-0.02 (-0.14 to 0.09)	0.688
***Bordetella pertussis***	21	2.09 (0.99)	0.43 (0.01 to 0.85)	0.043	0.40 (-0.01 to 0.82)	0.058	0.40 (0.00 to 0.79)	0.049
***Chlamydia pneumoniae***	11	1.99 (1.20)	0.33 (-0.36 to 1.01)	0.349	0.28 (-0.40 to 0.96)	0.421	0.36 (-0.30 to 1.01)	0.289
***Mycoplasma pneumoniae***	71	1.82 (1.10)	0.16 (-0.10 to 0.42)	0.230	0.13 (-0.14 to 0.40)	0.351	0.24 (-0.05 to 0.52)	0.103
**Group C beta-haemolytic Streptococci**	15	1.83 (0.86)	0.16 (-0.26 to 0.59)	0.453	0.13 (-0.30 to 0.55)	0.562	0.20 (-0.24 to 0.64)	0.376
**Group G beta-haemolytic Streptococci**	23	1.97 (1.18)	0.31 (-0.17 to 0.79)	0.204	0.28 (-0.20 to 0.76)	0.247	0.12 (-0.35 to 0.60)	0.609
***Staphylococcus aureus***	430	1.73 (1.07)	0.09 (-0.03 to 0.21)	0.161	0.07 (-0.06 to 0.20)	0.272	-0.02 (-0.14 to 0.11)	0.808
***Haemophilus influenzae***	314	1.68 (1.06)	0.01 (-0.12 to 0.15)	0.837	-0.03 (-0.18 to 0.12)	0.681	-0.02 (-0.16 to 0.13)	0.825
**Group A beta-haemolytic Streptococci**	100	1.65 (1.08)	-0.02 (-0.24 to 0.20)	0.850	-0.05 (-0.27 to 0.17)	0.645	-0.09 (-0.31 to 0.12)	0.403
***Streptococcus pneumoniae***	200	1.60 (0.99)	-0.08 (-0.23 to 0.07)	0.321	-0.14 (-0.31 to 0.02)	0.090	-0.11 (-0.27 to 0.06)	0.203
***Bordetella parapertussis***	11	1.20 (1.13)	-0.47 (-1.10 to 0.17)	0.149	-0.49 (-1.11 to 0.13)	0.123	-0.43 (-1.08 to 0.21)	0.190
***Moraxella catarrhalis***	4	0.89 (0.37)	-0.78 (-1.10 to -0.47)	<0.001	-0.80 (-1.10 to -0.50)	<0.001	-0.76 (-1.06 to -0.45)	<0.001

The reference group for analyses investigating the relationship between presence or absence of a single bacterium and mean symptom score was absence of the corresponding bacterium.

* Model adjusted for age, sex, ethnicity, index of multiple deprivation, clinician-reported baseline illness severity, duration of illness preceding presentation, presence of chronic disease, whether antibiotic prescribed at time of presentation, and presence of viruses. Models including individual bacteria were additionally adjusted for the presence of one or more other bacteria.

## Discussion

### Summary of main findings

This exploratory analysis is one of the first to demonstrate associations between the detection of respiratory viruses from the upper respiratory tract and prognosis in children recruited from primary care. We showed that the detection of three viruses (Influenza A, Influenza B, and RSV) and one bacterium (*Bordetella pertussis*) were potentially associated with higher symptom severity, and one virus (Enterovirus) and one bacterium (*Moraxella catarrhalis*) with lower symptom severity, between days 2–4 after illness presentation. We also observed that children testing positive for at least one virus, on average, were younger and presented to primary care more rapidly than those in whom no virus was detected, while the converse was true for children with any bacterium detected.

### Comparison with existing literature

We are aware of only two other studies investigating the prognostic significance of a range of upper respiratory tract microbes in primary care patients, both recruiting adults with acute lower respiratory tract infections [16,24]. The first [24] was a European study of 2,957 adults from whom one or more viruses were detected in 1,354 (46%) participants. In that study, those with RSV or Human Metapneumovirus detected had more prolonged symptoms compared to those with no virus detected. The second [16] was a study of 645 adults, which reported that symptom severity scores on days 2–4 were higher in patients in whom one or more viruses were detected, and those in whom both a virus and bacterium were detected, compared to those in whom no microbes were detected. A further UK study demonstrated that children aged 5–16 years presenting to primary care settings with a persistent cough and serological evidence of a recent *Bordetella pertussis* infection were more likely to experience severe and prolonged symptoms compared with those without evidence of *Bordetella pertussis* infection [25]. Similar studies in children [3] were conducted in secondary care settings and found that RSV, Adenovirus and Influenza were associated with longer times to hospital discharge following hospitalisation.

We recently demonstrated that microbiological POCT testing for multiple viruses and bacteria was acceptable to parents, patients and clinicians in the primary care setting [12]. Further, POCT testing led to increased diagnostic certainty and reduced expectation of antibiotic effectiveness in clinicians [12]. Only one other study evaluated the use of multiplex PCR point-of-care testing in a primary care setting, demonstrating modification of antibiotic prescribing [26]. Several other studies have investigated the use of POCT tests for the detection of influenza and RSV, again demonstrating a reduction in inappropriate antibiotic prescribing [27,28]. A Cochrane systematic review concluded that rapid, point‐of‐care antigen and molecular‐based tests with adequate accuracy may be considered an appropriate alternative to laboratory-based PCR tests when used to support timely patient care decisions [29]. POCTs testing for CRP and other biomarkers have also demonstrated some ability to discriminate between viral and bacterial RTIs [30] and to reduce inappropriate antibiotic prescribing [31] in primary care settings. However, none of these studies investigated the relationship between microbes detected and patient prognosis. Further, uptake of these biomarker-based approaches has been low and they are unable to definitively identify the aetiology of illness [32].

### Strengths and weaknesses

To our knowledge, this is the first study of its kind in children. The prospective design allowed us to measure and adjust for confounding variables, and we used a validated outcome measure [19]. The implementation of pharyngeal swabbing was acceptable to nearly all parents and children. Mean symptom severity scores were similar to those observed previously [33].

This study had several weaknesses. First, this was an exploratory, hypothesis-generating analysis and may therefore have been insufficiently powered to detect important relationships. It may also have been prone to type 1 error owing to multiple testing. Second, we took a pragmatic decision to use throat swabs for microbial detection. While this proved highly acceptable to parents and children, combined throat-nasal samples might have been preferable. These have been widely used during the SARS-CoV-2 pandemic [34]. Third, some samples were transported using Royal Mail postal services. Mean time between swabbing and laboratory arrival was previously found to be 2 days (range 1 to 21), with the likelihood of obtaining at least one positive (Ct≤35) result decreasing with increasing time (OR 0.94 95% CI 0.89 to 0.997 p = 0.038, *unpublished data*). Thus, we may have missed detecting some microbes of importance. We identified that those completing the symptom diaries were less deprived, as observed in previous prospective community-based studies [35,36]. Finally, symptom diary data was missing for a substantial proportion of children in the original study cohort, who were not included in this analysis. These latter weaknesses may limit the representativeness of the study cohort and generalisability of our findings.

### Implications

Establishing that some upper respiratory tract microbes may influence disease prognosis suggests that they could be playing an aetiological role. Microbiological POCT utilising a comparable approach [12] might therefore be useful in the management of infections in primary care. This would include improving the appropriate use of antibiotics and antivirals [37]. Further, it may help clinicians to provide more accurate predictions regarding illness trajectory, reduce clinical uncertainty, and provide increased safety netting where appropriate [18].

However, while there is promising evidence that microbiological POCTs are acceptable and may influence diagnostic reasoning [12], further research is needed before they are introduced into routine practice and policy recommendations. Much larger observational studies are needed to test: (i) if the findings of the present study can be replicated; (ii) if these relationships hold true for other microbes; (iii) if the relative changes in symptom severity (range 16% to 29%) are clinically important, and perhaps most importantly given the high cost of POCTs (although this is expected to decline over time); (iv) if the detection of upper respiratory tract microbes adds prognostic value to the information already available to clinicians–that is, symptoms and signs.

### Conclusion

This novel evidence, demonstrating that the detection of upper respiratory tract viruses, and less so bacteria, influences infection prognosis in children, suggests that there is a role for upper respiratory tract viral POCTs. Further, larger studies are needed to replicate these findings and explore any potential utility of microbiological POCTs in safely reducing antibiotic prescribing in primary care.

## Supporting information

S1 Fig Baseline data collection proforma. (TIF)

S1 Table Baseline characteristics of recruited children who were included or excluded from this study. *Observations with missing data, highest number for which was n = 7. † The English Indices of Multiple Deprivation 2007 score, linked to the child’s postcode, was used as a surrogate for home deprivation. ^‡^ Chronic diseases were defined as asthma, bronchiectasis, cystic fibrosis, diabetes, epilepsy, HIV, splenectomy, or ‘other’.
(DOCX)

S2 Table Baseline characteristics of children stratified by the presence of any bacteria detected. * The English Indices of Multiple Deprivation 2007 score, linked to the child’s postcode, was used as a surrogate for home deprivation. ^†^ Chronic diseases were defined as asthma, bronchiectasis, cystic fibrosis, diabetes, epilepsy, HIV, splenectomy, or ‘other’. ^a^ Viruses tested were Adenovirus, Bocavirus, Coronavirus, Enterovirus, Influenza A and B, Metapneumoviruses, Parainfluenzavirus types 1–4, Parechovirus, Respiratory Syncytial Virus (RSV), and Rhinovirus.
(DOCX)

S3 Table Age distribution of positive test results for the most commonly detected viruses and bacteria. Figures shown in brackets indicate the percentage of all children in the corresponding age group who tested positive for the corresponding microbe(s). * Types A and B. ^†^ Types 1–4.
(DOCX)
