# Supplementary figures and images for: Prognostic value of upper respiratory tract microbes in children presenting to primary care with respiratory infections: A prospective cohort study
Source: PLoS One. 2022 May 12;17(5):e0268131. doi: 10.1371/journal.pone.0268131 (PMC9098075; doi:10.1371/journal.pone.0268131)

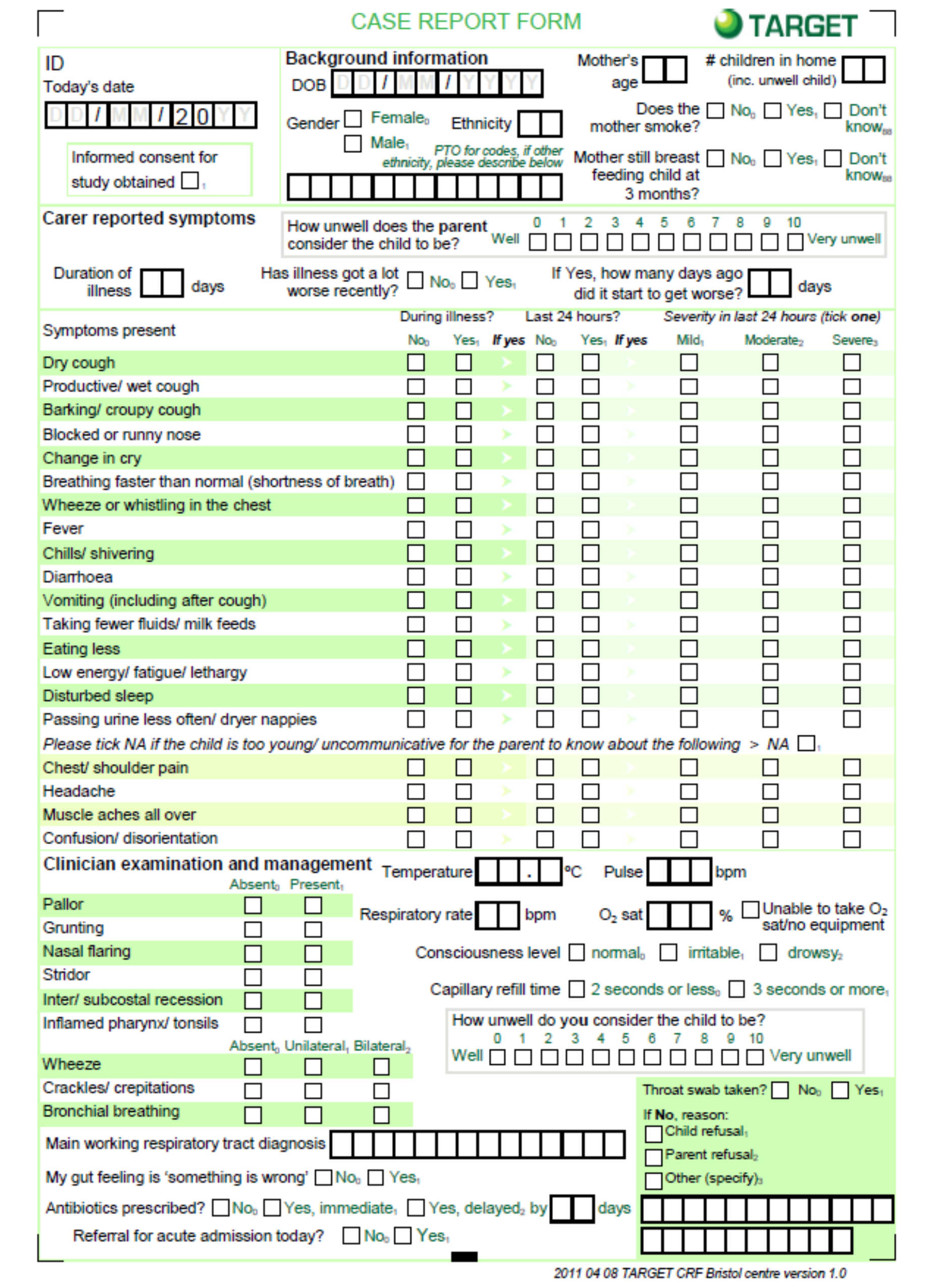

Supplement: S1 Fig — (TIF) [file pone.0268131.s001.tif]
