# Supplementary material for: Prognostic value of upper respiratory tract microbes in children presenting to primary care with respiratory infections: A prospective cohort study
Source: PLoS One. 2022 May 12;17(5):e0268131. doi: 10.1371/journal.pone.0268131 (PMC9098075; doi:10.1371/journal.pone.0268131)
Supplement: S1 Table — *Observations with missing data, highest number for which was n = 7. † The English Indices of Multiple Deprivation 2007 score, linked to the child’s postcode, was used as a surrogate for home deprivation. ‡ Chronic diseases were defined as asthma, bronchiectasis, cystic fibrosis, diabetes, epilepsy, HIV, splenectomy, or ‘other’. (DOCX) [file pone.0268131.s002.docx]

|  | **Included**  **(n = 1317)** | **Excluded**  **(n = 979)** |
| --- | --- | --- |
| **Mean age in years (SD)** | 4.80 (4.07) | 4.55 (3.90) |
| **Male, n (%)** | 694 (52.7) | 494 (50.5)* |
| **White, n (%)** | 1182 (89.7) | 836 (85.4)* |
| **IMD decile**†**, n (%)** |  |  |
| **Least deprived 1** | 133 (10.1) | 91 (9.5) |
| **2** | 159 (12.1) | 91 (9.5) |
| **3** | 105 (8.0) | 63 (6.6) |
| **4** | 135 (10.3) | 80 (8.3) |
| **5** | 132 (10.0) | 88 (9.2) |
| **6** | 158 (12.0) | 85 (8.9) |
| **7** | 114 (8.7) | 104 (10.8) |
| **8** | 122 (9.3) | 106 (11.0) |
| **9** | 129 (9.8) | 120 (12.5) |
| **Most deprived 10** | 130 (9.9) | 132 (13.8) |
| **History of chronic disease^‡^, n (%)** | 256 (19.4) | 182 (18.6) |
| **Median duration of illness before presentation (IQR)** | 5 (3-10) | 5 (3-9) |
| **Median clinician-reported illness severity at consultation (IQR)** | 3 (2-4) | 3 (2-4) |
| **Antibiotic prescribed at consultation, n (%)** | 509 (38.7) | 373 (39.2) |
| **Re-consultation within 30 days, n (%)** | 299 (22.7) | 232 (23.7)* |
| **Hospitalisation within 30 days, n (%)** | 18 (1.4) | 12 (1.2) |
| **Baseline Symptoms, n (%)** |  |  |
| Dry cough | 697 (52.9) | 515 (52.7) |
| Productive cough | 785 (59.6) | 585 (59.8) |
| Barking cough | 398 (30.2) | 280 (28.6) |
| Blocked/runny nose | 1013 (76.9) | 746 (76.4) |
| Change in cry | 240 (18.3) | 199 (20.4) |
| Shortness of breath | 546 (41.5) | 397 (40.6) |
| Wheeze | 590 (44.8) | 437 (44.7) |
| Fever | 794 (60.3) | 596 (60.9) |
| Shivering | 336 (25.5) | 252 (25.8) |
| Diarrhoea | 179 (13.6) | 150 (15.3) |
| Vomiting | 375 (28.5) | 309 (31.6) |
| Reduced fluid intake | 366 (27.8) | 317 (32.5) |
| Reduced eating | 797 (60.5) | 601 (61.5) |
| Low energy | 679 (51.6) | 478 (48.9) |
| Disturbed sleep | 1041 (79.0) | 757 (77.5) |
| Reduced urine output | 174 (13.2) | 135 (13.8) |
| **Baseline Clinical signs, n (%)** |  |  |
| Elevated pulse | 82 (6.3) | 57 (5.9) |
| Wheeze | 247 (18.8) | 121(21.0) |
| Crackles | 283 (21.5) | 218 (22.3) |
| Bronchial Breathing | 26 (2.0) | 31 (3.2) |
