# Supplementary material for: Prognostic value of upper respiratory tract microbes in children presenting to primary care with respiratory infections: A prospective cohort study
Source: PLoS One. 2022 May 12;17(5):e0268131. doi: 10.1371/journal.pone.0268131 (PMC9098075; doi:10.1371/journal.pone.0268131)
Supplement: S2 Table — * The English Indices of Multiple Deprivation 2007 score, linked to the child’s postcode, was used as a surrogate for home deprivation. † Chronic diseases were defined as asthma, bronchiectasis, cystic fibrosis, diabetes, epilepsy, HIV, splenectomy, or ‘other’. a Viruses tested were Adenovirus, Bocavirus, Coronavirus, Enterovirus, Influenza A and B, Metapneumoviruses, Parainfluenzavirus types 1–4, Parechovirus, Respiratory Syncytial Virus (RSV), and Rhinovirus. (DOCX) [file pone.0268131.s003.docx]

|  | **Bacteria**  **(n = 783)** | **No bacteria**  **(n = 534)** |
| --- | --- | --- |
| **Mean age (years, SD)** | 5.10 (4.14) | 4.36 (3.91) |
| **Male, n (%)** | 401 (51.2) | 293 (54.9) |
| **White, n (%)** | 698 (89.1) | 484 (90.6) |
| **IMD decile*, n (%)** |  |  |
| Least deprived 1 | 70 (8.9) | 63 (11.8) |
| 2 | 93 (11.9) | 66 (12.4) |
| 3 | 75 (9.6) | 30 (5.6) |
| 4 | 93 (11.9) | 42 (7.9) |
| 5 | 85 (10.9) | 47 (8.8) |
| 6 | 93 (11.9) | 65 (12.2) |
| 7 | 59 (7.5) | 55 (10.3) |
| 8 | 65 (8.3) | 57 (10.7) |
| 9 | 73 (9.3) | 56 (10.5) |
| Most deprived 10 | 77 (9.8) | 53 (9.9) |
| **History of chronic disease**^†^**, n (%)** |  |  |
| **Median number of days of illness before presentation (IQR)** | 6 (3-10) | 5 (3-10) |
| **Median clinician-reported Illness severity at consultation, score (IQR)** | 3 (2-4) | 3 (2-4) |
| **Antibiotic prescribed at consultation, n (%)** | 298 (38.1) | 211 (39.5) |
| **Re-consultation within 30 days, n (%)** | 189 (24.1) | 110 (20.6) |
| **Hospitalisation within 30 days, n (%)** | 10 (1.3) | 8 (1.5) |
| **Baseline symptoms, n (%)** |  |  |
| Dry cough | 425 (54.3) | 272 (50.9) |
| Productive cough | 466 (59.5) | 319 (59.7) |
| Barking cough | 234 (29.9) | 164 (30.7) |
| Blocked/runny nose | 608 (77.7) | 405 (75.8) |
| Change in cry | 137 (17.5) | 103 (19.4) |
| Shortness of breath | 337 (43.0) | 209 (39.1) |
| Wheeze | 350 (44.7) | 240 (45.0) |
| Fever | 481 (61.4) | 313 (58.6) |
| Shivering | 215 (27.5) | 121 (22.7) |
| Diarrhoea | 95 (12.1) | 84 (15.7) |
| Vomiting | 205 (26.2) | 170 (31.8) |
| Reduced fluid intake | 218 (27.9) | 148 (27.7) |
| Reduced eating | 475 (60.7) | 322 (60.3) |
| Low energy | 428 (54.7) | 251 (47.0) |
| Disturbed sleep | 628 (80.2) | 413 (77.3) |
| Reduced urine output | 106 (13.6) | 68 (12.7) |
| **Baseline clinical signs, n (%)** |  |  |
| Elevated pulse | 47 (6.1) | 35 (6.6) |
| Wheeze | 142 (18.1) | 105 (19.7) |
| Crackles | 156 (19.9) | 127 (23.8) |
| Bronchial Breathing | 18 (2.3) | 8 (1.5) |
| **Number of bacteria present, n (%)** |  |  |
| 1 | 481 (61.4) | - |
| 2 | 225 (28.7) | - |
| 3 | 62 (7.9) | - |
| 4 | 15 (1.9) | - |
| **Number positive for individual bacteria, n (%)** |  |  |
| *Staphylococcus aureus* | 430 (54.9) | - |
| *Haemophilus influenzae* | 314 (40.1) | - |
| *Streptococcus pneumoniae* | 200 (25.5) | - |
| Group A beta-haemolytic Streptococci | 100 (12.8) | - |
| *Mycoplasma pneumoniae* | 71 (9.1) | - |
| Group G beta-haemolytic Streptococci | 23 (2.9) | - |
| *Bordetella pertussis* | 21 (2.7) | - |
| Group C beta-haemolytic Streptococci | 15 (1.9) | - |
| *Chlamydia pneumoniae* | 11 (1.4) | - |
| *Bordetella parapertussis* | 11 (1.4) | - |
| *Moraxella catarrhalis* | 4 (0.5) | - |
| Group F beta-haemolytic Streptococci | 0 (0) | - |
| **Viruses^a^ detected, n (%)** | 548 (70.0) | 321 (60.1) |
