# Supplementary material for: Prognostic value of upper respiratory tract microbes in children presenting to primary care with respiratory infections: A prospective cohort study
Source: PLoS One. 2022 May 12;17(5):e0268131. doi: 10.1371/journal.pone.0268131 (PMC9098075; doi:10.1371/journal.pone.0268131)
Supplement: S3 Table — Figures shown in brackets indicate the percentage of all children in the corresponding age group who tested positive for the corresponding microbe(s). * Types A and B. † Types 1–4. (DOCX) [file pone.0268131.s004.docx]

|  | **<1 year** | **1-2 years** | **3-4 years** | **4-5 years** | **6-9 years** | **≥10 years** | **Overall** |
| --- | --- | --- | --- | --- | --- | --- | --- |
| **≥1 virus** | 156 (70.3) | 166 (81.4) | 211 (69.6) | 128 (65) | 93 (56.4) | 98 (53.3) | 852 (66.8) |
| Rhinovirus | 74 (33.6) | 62 (30.9) | 82 (27.6) | 54 (27.8) | 42 (26.3) | 39 (21.4) | 353 (28.2) |
| RSV* | 36 (16.3) | 37 (18.2) | 35 (11.6) | 14 (7.1) | 6 (3.7) | 6 (3.3) | 134 (10.6) |
| Enterovirus | 19 (8.8) | 41 (20.5) | 31 (10.5) | 17 (9) | 7 (4.4) | 8 (4.4) | 123 (9.9) |
| Parainfluenzae^†^ | 23 (10.4) | 27 (13.2) | 35 (11.6) | 17 (8.6) | 7 (4.2) | 9 (4.9) | 118 (9.3) |
| Influenza A or B | 5 (2.3) | 11 (5.4) | 26 (8.6) | 26 (13.2) | 31 (18.8) | 31 (16.9) | 130 (10.2) |
| **≥1 bacterium** | 121 (54.5) | 121 (59.3) | 158 (52.2) | 118 (59.9) | 113 (68.5) | 120 (65.2) | 751 (58.9) |
| *Staphylococcus aureus* | 84 (37.8) | 50 (24.5) | 61 (20.2) | 50 (25.4) | 75 (45.7) | 94 (51.4) | 414 (32.6) |
| *Haemophilus influenzae* | 39 (17.6) | 61 (29.9) | 80 (26.5) | 51 (25.9) | 48 (29.3) | 29 (15.9) | 308 (24.2) |
| *Streptococcus pneumoniae* | 39 (17.6) | 46 (22.6) | 49 (16.2) | 25 (12.7) | 18 (11) | 17 (9.3) | 194 (15.3) |
| Group A beta-haemolytic streptococci | 3 (1.4) | 10 (4.9) | 21 (7) | 29 (14.7) | 25 (15.2) | 7 (3.8) | 95 (7.5) |
